# Stress, depression, and risk of dementia – a cohort study in the total population between 18 and 65 years old in Region Stockholm

**DOI:** 10.1186/s13195-023-01308-4

**Published:** 2023-10-02

**Authors:** Johanna Wallensten, Gunnar Ljunggren, Anna Nager, Caroline Wachtler, Nenad Bogdanovic, Predrag Petrovic, Axel C. Carlsson

**Affiliations:** 1 Department of Clinical Sciences, Danderyd Hospital, 18288 Stockholm, Sweden; 2 Academic Primary Health Care Centre, Solnavägen 1E, 104 31 Stockholm, Sweden; 3 Division of Family Medicine and Primary Health Care, Department of Neurobiology, Care Sciences and Society, Karolinska Institutet, 17177 Stockholm, Sweden; 4 Center for Cognitive Psychiatry, Department of Clinical Neuroscience, Karolinska Institutet, Stockholm, Sweden; 5 Center for Cognitive and Computational Neurosceince (CCNP), Karolinska Institutet, Stockholm, Sweden

**Keywords:** Administrative databases, General population, Mental illness, Psychiatric disorders, Stress, Stress-related disorder, Depression, Dementia, Alzheimer, Neurodegenerative disorders

## Abstract

**Background:**

Chronic stress and depression are potential risk factors for mild cognitive impairment and dementia, including Alzheimer disease. The aim was to investigate whether any such risk is additive.

**Methods:**

Cohort study including 1 362 548 people (665 997 women, 696 551 men) with records in the Region Stockholm administrative healthcare database (VAL).

Exposure was a recorded ICD-10 diagnosis of chronic stress, depression, or both, recorded in 2012 or 2013. Outcome was a diagnosis of Alzheimer disease, other dementia, or mild cognitive impairment recorded from 2014 through 2022. Odds ratios with 99% confidence intervals (CI) adjusted for age, sex, neighborhood socioeconomic status, diabetes, and cardiovascular disorders were calculated.

**Results:**

During the exposure period, 4 346 patients were diagnosed with chronic stress, 40 101 with depression, and 1 898 with both. The average age at baseline was around 40 years in all groups. In the fully adjusted model, the odds ratio of Alzheimer disease was 2.45 (99% CI 1.22–4.91) in patients with chronic stress, 2.32 (99% CI 1.85–2.90) in patients with depression, and 4.00 (99% CI 1.67–9.58) in patients with chronic stress and depression. The odds ratio of mild cognitive impairment was 1.87 (99% CI 1.20–2.91) in patients with chronic stress, 2.85 (99% CI 2.53–3.22) in patients with depression, and 3.87 (99% CI 2.39–6.27) in patients with both. When other dementia was analyzed, the odds ratio was significant only in patients with depression, 2.39 (99% CI 1.92–2.96).

**Conclusions:**

Documented chronic stress increased the risk of mild cognitive impairment and Alzheimer disease. The same was seen with depression. The novel finding is the potential additive effect of chronic stress to depression, on risk of MCI and AD.

## Background

Dementia affects more than 55 million people globally and is one of the most burdensome neurological disorders [22]. It is also one of the ten leading causes of death [1]. Alzheimer disease (AD) constitutes more than half of all dementia [1], and one-third of patients with mild cognitive impairment (MCI) develop AD within five years [15, 63]. Brain changes in AD include degeneration of nerve cells and accumulation of beta amyloid outside the neurons and phosphorylated tau inside the neurons.

Age is an important risk factor for dementia, which suggests that as the population ages, the current number of 55 million people with dementia may more than double by 2050. As age is not modifiable, prevention must focus on other known risk factors. A risk score model for dementia has identified predictors of increased risk in men and women [49]. The model includes excessive daytime sleepiness [13] and comorbidities such as respiratory disorders, diabetes, cerebrovascular disorders, and hypertension [49]. Some of these factors are modifiable, and some cases of dementia might therefor be preventable [39, 44].

Depression and chronic stress are other potentially modifiable risk factors for dementia [29, 39, 41, 51, 56]. Depression is common worldwide and a primary contributor to the global burden of disease [23, 26]. Research suggests that stress can contribute to the development of depression [42]. Chronic stress may also increase the risk for dementia via raised risk for depression, cardiovascular disorders, stroke, and autoimmune disorders [57] and possibly even through a direct causal mechanism [29].

Exposure to chronic, non-traumatic stress is difficult to measure. Self-rated stress scales often capture only a short exposure interval. Stress-related diagnoses such as adjustment disorder or post-traumatic stress disorder capture exposure to stress but not to longer-term non-trauma stress. The Swedish medical system includes a novel diagnosis that can be used as a proxy for such stress. This diagnosis, chronic stress-induced exhaustion disorder or SED (F43.8 in the Swedish ICD-10), is a condition caused by more than 6 months of intensive stress without sufficient recovery. Symptoms include exhaustion, sleep disturbance, and cognitive symptoms such as concentration difficulties and impaired memory [38].

Although previous epidemiological and mechanistic research suggests that both stress and depression are related to development of dementia, not much is known about whether they are part of the same mechanistic pathway or independently contribute to dementia risk. Moreover, AD with familial linkage or genetic polymorphism is dominant in younger people who develop dementia. These individuals are already vulnerable, and stress and depression can be the first symptoms of dementia.

We hypothesize that depression and stress are additive and that both contribute to the risk of developing dementia. The aim of the study was to investigate whether chronic stress and depression are associated with a higher risk for mild cognitive impairment (MCI) or dementia, including Alzheimer disease (AD), and whether any such risk is additive.

## Methods

### Study design and setting

This was a longitudinal cohort study of MCI and dementia, including AD, in men and women in the Region Stockholm administrative database (VAL) who were diagnosed with SED, depression, or both SED and depression between 2012 and 2013. Their risk of MCI and dementia was compared to the risk in men and women not diagnosed with SED or depression during the same period. The study population was followed for diagnoses of MCI or dementia between 2014 and 2022.

Region Stockholm is an administrative entity with the primary responsibility for publicly funded healthcare in a geographical area also called Region Stockholm. More than one-fifth of Sweden’s population, i.e., more than 2.2 million people, live in this area, which includes the capital city, Stockholm, as well as the surrounding rural area, a large archipelago, suburbs, and nearby towns. Almost all primary care, specialist outpatient care, and inpatient care diagnoses, drug prescriptions, and consultations are recorded in the central regional database, the Stockholm Regional Health Care Data Warehouse (VAL). The VAL-database enables prevalence and incidence studies of different diagnoses for all residents in the region [11]. Diagnoses are coded in accordance with the Swedish version of WHO’s International Classification of Diseases, 10th edition, ICD-10-SE, which includes the SED diagnosis. The information in the VAL-database is used to update Region Stockholm’s information in the Swedish National Patient Register, which is maintained by the National Board of Health and Welfare. The VAL- database has been almost entirely complete since 2007, with the exception of data from some privately funded clinics [65].

### Participants

The study cohort included men and women who resided in Region Stockholm between 1 January 2011 and 28 February 2022 and were between the ages of 18 and 65 years during the first year of inclusion.

All new diagnoses of SED, depression, or both SED and depression that were registered in patients’ medical records between 1 January 2012 and 31 December 2013 were included in the analyses. Patients with a recorded diagnoses of SED or depression in 2011 (the washout period) were excluded from the study. Men and women registered in the VAL-database without a diagnosis of SED or depression between 1 January 2012 and 31 December 2013 were used as the reference group. Patients with a recorded diagnosis of MCI, Alzheimer disease or other dementia recorded in 2012 or 2013 were excluded. Diagnoses of MCI, AD, and other dementia were collected from 2014 through 2022.

### Variables, measurement, and data sources

#### Sociodemography

The geodemographic profiling tool, Experian’s Mosaic Public Sector 6 (MPS6) © Experian 2014 [21] was used to classify socioeconomic status. The MPS6 uses more than 400 variables to classify small area codes into groups by socioeconomic status. MPS6 was primarily designed to help businesses in the United Kingdom target their services and products to the appropriate groups but has now been used in research in almost 30 countries [20, 54].

#### Diagnoses

ICD-10-SE diagnoses were used in this study, as diagnoses in the VAL-database are coded in accordance with this Swedish version of WHO’s International Classification of Diseases, 10th edition (ICD-10). Diagnoses registered after a consultation with a doctor were chosen.

Depression was defined as a diagnosis of ICD-10-SE codes F32 (depressive episode) through F33 (recurrent depressive disorder), and chronic stress as a diagnosis of SED (ICD-10-SE code F43.8). Alzheimer disease was defined as ICD-10-SE code F00 or G30, other dementia (including Lewy body, vascular, and mixed) as F01 through F03, and MCI as F06.7. The diagnostic process for investigating cognitive impairment starts in primary care settings involving comprehensive medical assessment, including symptom inventory and medical history/family history, clinical examination and cognitive screening, laboratory tests and imaging. In cases under 65 years old, where a dementia diagnosis is suspected, the patient is referred for extensive neuropsychological testing and CSF analysis alongside a structured evaluation of functional and activity capacity in a special unit for dementia. The investigation includes lumbar puncture and analysis of AD markers, as well as IgG production as part of the baseline assessment. Thus, the clinical assessment leading to a dementia diagnosis is thorough and reliable in this population. Patients with a recorded diagnosis of MCI, Alzheimer disease or other dementia recorded in 2012 or 2013 were excluded.

Covariates included diabetes mellitus (ICD-10-SE codes E10, E11, and E14) and cardiovascular disorders, including hypertensive disease, ischemic heart disease, heart failure, stroke, transient ischemic attack, and peripheral vascular disease (ICD-10-SE codes I10–13, I15, I20–25, I50, 60–69, and I73).

### Statistical analysis

Logistic regression was used to calculate odds ratios (ORs) of MCI, AD, and other dementia (diagnosed between 2014 and 2022) in patients whose medical record included chronic stress, depression, or both diagnosed between 2012 and 2013. The reference group consisted of patients whose medical record did not include a diagnosis of chronic stress or depression between 2012 and 2013. The logistic regression models were first a crude model, then a model adjusted for age, sex, and neighborhood socioeconomic status. In the fully adjusted logistic regression models diabetes mellitus and cardiovascular disorders were added. In logistic regression, we chose to report odds ratios when 4 or more observations were present.

To decrease the risk of significant results due to multiple testing, a p-value of less than 0.01 was considered significant, and a 99% confidence interval (CI) was used. Confidence intervals that did not include 1 were considered significant.

SAS version 9.4 (SAS Institute Inc., Cary, NC) was used for the statistical analyses.

## Results

**Fig. 1:**
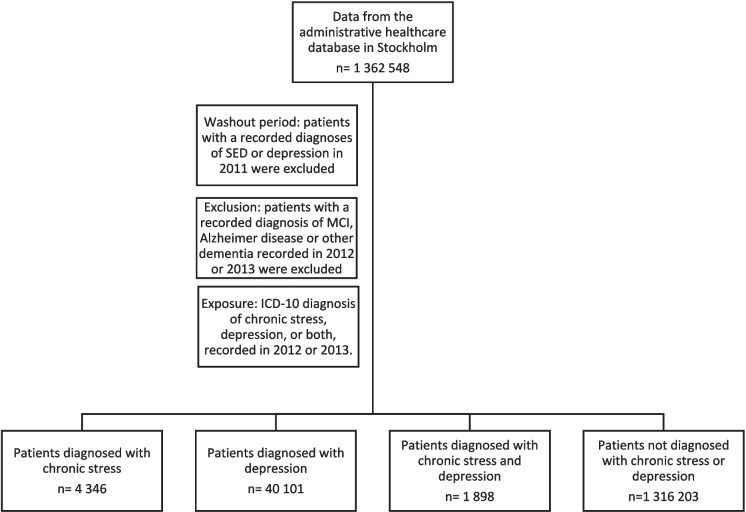
Flowchart of inclusion from the administrative healthcare database in Stockholm. Abbreviations: SED, chronic stress-induced exhaustion, MCI, Mild cognitive impairment

Descriptive characteristics of the “Region Stockholm cohort” (n = 1 362 548) are shown in Table 1. During 2012–2013 a diagnosis of SED was recorded in 4 346 patients (0.3%), a diagnosis of depression was recorded in 40 101 patients (2.9%) and a diagnosis of both SED and depression in 1 898 patients (0.1%). In 1316 203 people (96.6%) no diagnosis of SED or depression was recorded (Fig. 1).

**Table 1:** Characteristics of the total population between 18 and 65 years of age in Region Stockholm by presence of chronic stress-induced exhaustion (SED), depression (DEP), or both as well as referents without registered diagnoses of SED or DEP

	**All (n = 1 362 548)**				**Women (n = 665 997)**				**Men (n = 696 551)**			
	**SED**	**DEP**	**SED + DEP**	**No SED/ DEP**	**SED**	**DEP**	**SED + DEP**	**No SED/ DEP**	**SED**	**DEP**	**SED + DEP**	**No SED/ DEP**
**Baseline (2012–2013)**												
n (% of the total study population)	4 346 (0.3)	40 101 (2.9)	1 898 (0.1)	1 316 203 (96.6)	3 368 (0.5)	25 854 (3.9)	1497 (0.2)	635 278 (95.4)	978 (0.1)	14 247 (2.0)	401 (0.1)	680 925 (97.8)
Age years mean (median)	42.05 (42.00)	38.60 (38.00)	41.74 (41.00)	39.69 (39.00)	42.10 (42.00)	38.44 (37.00)	41.63 (41.00)	39.74 (39.00)	41.90 (42.00)	38.90 (38.00)	42.16 (42.00)	39.65 (39.00)
**Diagnoses (2014–2022)**												
Alzheimer disease,^a^ n (%) Age years mean (median) (range)	14 (0.32) 57.29 (57.00) (52–64)	148 (0.37) 60.09 (62.00) (39–65)	9 (0.47) 54.89 (55.00) (40–61)	2 557 (0.19) 60.38 (62.00) (29–65)	13	103	8	1 438	1	45	1	1 119
Other dementia,^a^ n (%) Age years mean (median) (range)	5 (0.12) 56.60 (57.00) (51–62)	160 (0.40) 58.13 (60.00) (21–65)	3 (0.16) 57.00 (58.00) (53–60)	2 654 (0.20) 59.38 (61.00) (18–65)	4	81	3	1 093	1	79	0	1 561
Mild cognitive disorder,^c^ n (%) Age years mean (median) (range)	36 (0.83) 52.89 (54.50) (37–63)	528 (1.32) 54.02 57.00 (18–65)	31 (1.63) 48.26 50.00 (22–61)	6 754 (0.51) 56.13 60.00 (18–65)	32	327	24	3 273	4	201	7	3 481
Diabetes mellitus,^d^ n (%)	234 (5.38)	3 153 (7.86)	126 (6.64)	81 890 (6.22)	161	1714	85	30 391	73	1439	41	51 499
Other cardiovascular disease,^e^ n (%)	986 (22.69)	9 298 (23.19)	446 (23.50)	269 363 (20.47)	725	5403	324	119 658	261	3895	122	149 705

Abbreviations: SED, chronic stress-induced exhaustion, DEP, depression

^a^ICD-10 code F00 Dementia in Alzheimer disease and G30 Alzheimer disease

^b^ICD-10 code F01 Vascular dementia, F02 Dementia in other diseases classified elsewhere, and F03 Unspecified dementia

^c^ICD-10 code F06.7 Mild cognitive impairment

^d^ICD-10 code E10 Type 1 diabetes mellitus, E11 Type 2 diabetes mellitus, and E14 Unspecified diabetes mellitus

^e^ICD-10 code I10 Essential (primary) hypertension, I11 Hypertensive heart disease, I12 Hypertensive renal disease, I13 Hypertensive heart and renal disease, I15 Secondary hypertension

I20-25 Ischaemic heart diseases, I50 Heart failure, I60-69 Cerebrovascular diseases, and I73 Other peripheral vascular diseases

Recorded diagnoses of AD, other dementia, and MCI during 2014–2022 are shown in Table 1 as well as recorded diagnoses of diabetes mellitus and other cardiovascular disease.

Associations between receiving a diagnosis of SED, depression, or both, in 2012–2013, and AD, other dementias, or MCI between 2014–2022 are shown in Table 2. The model was a crude model without adjustments. The same association is shown in Table 3. This model was adjusted for age, sex and neighborhood socioeconomic status. In Table 4 the models were adjusted for age, sex, neighborhood socioeconomic status, diabetes mellitus and cardiovascular disorders. The models found in Tables 2, 3 and 4 are stratified by sex. In the model adjusted for age, sex, neighborhood socioeconomic status, diabetes mellitus and cardiovascular disorder (Table 4) the OR for AD was 2.45 (99% CI 1.22–4.91) in patients with SED, 2.32 (99% CI 1.85–2.90) in patients with depression, and 4.00 (99% CI 1.67–9.58) in patients with both SED and depression. The OR for MCI was 1.87 (99% CI 1.20–2.91) in patients with SED, 2.85 (99% CI 2.53–3.22) in patients with depression, and 3.87 (99% CI 2.39–6.27) in patients with both. In patients with other dementia, the OR was significant only in patients with depression 2.39 (99% CI 1.92–2.96).

**Table 2:** Logistic regression models for the association between registered diagnoses of chronic stress-induced exhaustion (SED), depression (DEP), or both in 2012 or 2013 and the outcomes Alzheimer disease, other dementia, or mild cognitive disorder between 2014 and 2022. Crude model without adjustments

**Diagnosis**		**All**			**Women**			**Men**		
	**No SED/ DEP**	**SED**	**DEP**	**SED + DEP**	**SED**	**DEP**	**SED + DEP**	**SED**	**DEP**	**SED + DEP**
Alzheimer disease,^a^ OR (99% CI)	1.00	1.66 (0.83–3.32)	1.90 (1.53–2.37)	2.45 (1.03–5.80)	1.71 (0.83–3.51)	1.76 (1.36–2.29)	2.37 (0.95–5.92)	NA	1.93 (1.30–2.85)	NA
Other dementia,^b^ OR (99% CI)	1.00	0.57 (0.18–1.81)	1.98 (1.61–2.45)	0.78 (0.18–3.47)	0.69 (0.19–2.51)	1.82 (1.36–2.46)	NA	NA	2.43 (1.80–3.27)	NA
Mild cognitive impairment,^c^ OR (99% CI)	1.00	1.62 (1.05–2.50)	2.59 (2.30–2.91)	3.22 (2.02–5.14)	1.85 (1.17–2.93)	2.47 (2.13–2.88)	3.15 (1.85–5.36)	0.80 (0.22–2.91)	2.79 (2.31–3.36)	3.47 (1.30–9.25)

*Abbreviations*: *DEP* Depression, *NA* Too few obeservations to calculate a reliable risk estimate, *SED* Chronic stress-induced exhaustion

^a^ICD-10 code F00 Dementia in Alzheimer disease and G30 Alzheimer disease

^b^ICD-10 code F01 Vascular dementia, F02 Dementia in other diseases classified elsewhere, and F03 Unspecified dementia

^c^ICD-10 code F06.7 Mild cognitive impairment

**Table 3:** Logistic regression models for the association between registered diagnoses of chronic stress-induced exhaustion (SED), depression (DEP), or both in 2012 or 2013 and the outcomes Alzheimer disease, other dementia, or mild cognitive disorder between 2014 and 2022 with adjustments for age, sex and neighborhood socioeconomic status

**Diagnosis**		**All**			**Women**			**Men**		
	**No SED/ DEP**	**SED**	**DEP**	**SED + DEP**	**SED**	**DEP**	**SED + DEP**	**SED**	**DEP**	**SED + DEP**
Alzheimer disease,^a^ OR (99% CI)	1.00	2.45 (1.22–4.92)	2.34 (1.87–2.92)	4.02 (1.68–9.61)	2.84 (1.38–5.88)	2.33 (1.79–3.05)	4.49 (1.77–11.37)	NA	2.35 (1.58–3.51)	NA
Other dementia,^b^ OR (99% CI)	1.00	0.92 (0.29–2.92)	2.51 (2.03–3.12)	1.37 (0.31–6.12)	1.03 (0.28–3.74)	2.23 (1.65–3.02)	NA	NA	2.88 (2.12–3.90)	NA
Mild cognitive impairment,^c^ OR (99% CI)	1.00	1.89 (1.21–2.94)	3.02 (2.68–3.41)	3.95 (2.44–6.40)	2.13 (1.33–3.41)	2.90 (2.49–3.38)	3.78 (2.18–6.54)	0.94 (0.26–3.42)	3.18 (2.62–3.85)	4.22 (1.55–11.43)

*Abbreviations*: *DEP* Depression, *NA* Too few obeservations to calculate a reliable risk estimate, *SED* Chronic stress-induced exhaustion

^a^ICD-10 code F00 Dementia in Alzheimer disease and G30 Alzheimer disease

^b^ICD-10 code F01 Vascular dementia, F02 Dementia in other diseases classified elsewhere, and F03 Unspecified dementia

^c^ICD-10 code F06.7 Mild cognitive impairment

**Table 4:** Logistic regression models for the association between registered diagnoses of chronic stress-induced exhaustion (SED), depression (DEP), or both in 2012 or 2013 and the outcomes with adjustment for age, sex, neighborhood socioeconomic status, diabetes mellitus and cardiovascular disorder

**Diagnosis**		**All**			**Women**			**Men**		
	**No SED/ DEP**	**SED**	**DEP**	**SED + DEP**	**SED**	**DEP**	**SED + DEP**	**SED**	**DEP**	**SED + DEP**
Alzheimer disease,^a^ OR (99% CI)	1.00	2.45 (1.22–4.91)	2.32 (1.85–2.90)	4.00 (1.67–9.58)	2.81 (1.37–5.88)	2.32 (1.77–3.04)	4.48 (1.77–11.34)	NA	2.32 (1.56–3.47))	NA
Other dementia,^b^ OR (99% CI)	1.00	0.92 (0.29–2.91)	2.39 (1.92–2.96)	1.36 (0.31–6.06)	1.02 (0.28–3.73)	2.11 (1.56–2.86)	NA	NA	2.73 (2.01–3.70)	NA
Mild cognitive disorder,^c^ OR (99% CI)	1.00	1.87 (1.20–2.91)	2.85 (2.53–3.22)	3.87 (2.39–6.27)	2.12 (1.32–3.40)	2.76 (2.37–3.22)	3.72 (2.14–6.45)	0.93 (0.25–3.40)	2.97 (2.44–3.60)	4.12 (1.52–11.18)

*Abbreviations*: *DEP* Depression, *NA* Too few obeservations to calculate a reliable risk estimate, *SED* Chronic stress-induced exhaustion

^a^ICD-10 code F00 Dementia in Alzheimer disease and G30 Alzheimer disease

^b^ICD-10 code F01 Vascular dementia, F02 Dementia in other diseases classified elsewhere, and F03 Unspecified dementia

^c^ICD-10 code F06.7 Mild cognitive disorder

The results from the model adjusted for age, sex, neighborhood socioeconomic status (Table 3) were similar to results from the model adjusted for age, sex, neighborhood socioeconomic status, diabetes mellitus and cardiovascular disorder (Table 4). The models were more robust in women due to larger number of female patients in all patient groups (patients with SED, patients with depression, patients with SED and depression).

## Discussion

### Main findings

The present study suggests an increased risk for MCI and AD if a patient with depression is additionally exposed to chronic stress as indicated by the diagnosis SED. Both preceding depression and SED were independently associated with increased risk for MCI and AD, but only depression was associated with increased risk for other dementia i.e., Lewy body, vascular and mixed dementia. However, as there were only a few cases, this association needs confirmation. The marked additional effect of chronic stress in patients with depression for developing MCI or dementia has, as far as we know, not previously been presented in an epidemiological cohort study.

Our findings are in line with previous findings that patients with depression [14, 24, 34] and patients exposed to stress [9, 29, 30, 64] seem to have an increased risk for MCI and dementia.

Several possible pathophysiological mechanisms have been suggested to explain how chronic stress may cause dementia. Chronic stress is a suggested trigger for continuum into AD [6]. The continuum consists of the pre-clinical phase, MCI, and AD. In the pre-clinical phase, the patient can report subjective cognitive difficulties, but cognitive testing does not show any decline, i.e., the brain can compensate for the brain changes, but biomarkers can be found in cerebrospinal fluid (CSF) and plasma [46]. When the brain cannot compensate for changes in the brain anymore, symptoms of MCI arise. When damage of neurons continues, symptoms start to interfere with daily life and the disease continue from mild to moderate and finally to severe dementia ("2022 Alzheimer's disease facts and figures," 2022). Chronic stress can affect the brain in different ways, activation of the stress axis (hypothalamus-pituitary and adrenal axis) increase the levels of cortisol that can cross the blood–brain barrier and impact amygdala, hippocampus and prefrontal cortex through specific receptors [5]. Also, corticotropin-releasing hormone acts directly on these circuits and is involved both in the development of depression [40] and dementia [28]. Chronic stress may accelerate the deposition of beta-amyloid plaque and hyperphosphorylation of tau and drive the pathogenesis behind AD, at least in mouse models [12, 58]. Additionally, research indicates that inflammatory processes may be involved in the pathophysiology of SED, depression, and neurodegenerative disorders such as AD [3, 35]. Stress can activate inflammation in the brain, which possibly could affect the permeability of the blood–brain barrier, but also affect the brain's nerves and supporting cells, such as astrocytes [62]. Astrocytes not only support the function of neurons but are also involved in the immune response. Chronic stress also increases the levels of pro-inflammatory cytokines that can affect neural activity [27]. Inflammation is a suggested link between early stress and later neurodegeneration [17]. Stress also affects risk factors such as cardiovascular factors and diabetes, and could therefore potentially increase the risk for dementia [31]. Another aspect is that dementia symptoms may increase stress and therefore the risk for depression. Accordingly, the short time between exposure to SED and depression and the outcome might reflect the experience of chronic stress and depressive symptoms being prodromal symptoms of MCI and dementia. The relationship between the timing of stress and the onset of dementia is a critical factor to consider in future longitudinal studies when trying to establish a causal link. The alterations in the brain associated with Alzheimer's disease may initiate up to 20 years prior to the manifestation of the first symptoms of memory impairment [32]. However, stress management may still be beneficial for cognition [41].

Previous research suggests a causal relationship between depression and dementia. Depression has been found to be associated with dementia [33], however, it may both increase the risk of dementia [34], but also be causal or prodromal [8]. Depression and dementia also share risk factors, such as older age, low socioeconomic or educational level, comorbidity, decreased activity, and poor health as well as some common biomarkers such as tau protein [7]. Depression is also linked to chronic low grade inflammation, ie, another possible link to the pathophysiology behind AD [16]. Moreover, hippocampus is a primary locus in both AD and depression, however, different subfields are affected [47].

Our data suggest that chronic stress potentiates the risk for dementia related to depression. However, it is not known how stress and depression might contribute with additive effect on risk for dementia. In previous research, astrocytes as well as inflammation seem to be involved in the pathogenesis behind both SED [61, 62] depression [37] and dementia [18, 35]. Depression and chronic stress could therefore potentially be additive, resulting in an increased risk for MCI and dementia. Additionally, impaired sleep and cognitive symptoms are present in patients with both depression and chronic stress and could be other potential pathways [48, 55]. Impaired sleep decreases the function of the glymphatic system, i.e., increasing the levels of beta-amyloid in the brain due to impaired clearance [66].

There are other important factors that might be involved. In younger individuals who develop dementia, AD dominates with familial linkage or genetic polymorphism, these patients are already vulnerable [19], and stress and depression can be the first symptomatology of dementia. Alternatively, they may be affected more by stress and depression that speed up the pathological processes leading to dementia. Additionally, the APOE gene that is suggested to be involved in amyloid-beta metabolism, neuroinflammation, tau- induced neurodegeneration, and blood–brain barrier disruption, could affect the age of onset of cognitive impairment [53]. Vitamin D deficiency has been associated with cognitive symptoms and suggested involved in dementia and mood disorders [4, 43, 50]. Also, these factors may interact with stress and depression and further increase the risk for dementia.

### Strengths and limitations

The 2.9% prevalence of depression observed in this study is lower than the prevalence observed in survey-based research in Swedish primary care, which has found that approximately 6% of patients in waiting rooms report depressive symptoms severe enough to meet the criteria for clinical depression [45]. However, it is consistent with earlier findings based on clinically diagnosed depression in Swedish primary care. For instance, a study based on data from several areas in Sweden, including but not limited to Region Stockholm, found 2.4% of primary health care patients had a diagnosis of depression in 2002 [36]. Thus, the present study likely covers most people diagnosed with depression in primary care in the region but not all people with depressive symptoms. It is not clear how the inclusion of more people with depressive symptoms would have affected the results.

The diagnoses in this large clinical dataset are recorded according to clinical practice, which may differ from the requirements used for diagnoses in clinical trials. This can result in both over- and under-estimations of the prevalence. However, when the diagnoses were recorded, this study was not planned and could therefore not be responsible for a possible under- or over-estimation. However, in a register-based study from Denmark, a diagnosis of dementia in patients under 65 years were correct only in 59% [52]. According to that, we had access to registered diagnoses, all first-time diagnoses are registered by an experienced geriatrician or neurologist. However, future studies should analyze whether dementia proceeded by stress or/and depression also have been confirmed by CSF biomarkers.

Another limitation of using register data is that recorded diagnoses not always reflect diagnoses made by structured psychiatric interviews [2]. Due to small numbers of the outcome, data does not allow us to adjust for two health care visits that are measured in the patient group with SED and depression. This group might reflect not an additive effect of stress to depression, but a more severe illness, or possibly recurrent depression compared to patients with only SED or only depression. Except for AD, we did not differentiate between different dementia in the group with “other dementia”.

Our study design ensures that included patients with SED and depression did not have any diagnosis of SED or depression during 2011; however, we do not know if they have had any of these diagnoses prior to 2011.

Self-reported symptoms of psychological distress have been found to be associated with increased risk of dementia [59], but findings diverge. In patients with amnestic mild cognitive impairment, no association was found between adverse cognitive outcome and objective stress measures which include adverse life events, and subjective measures which include perceived stress or distress measures [60]. The SED diagnosis was used to enable an investigation of the impact of chronic stress on developing MCI or dementia. By using the diagnosis SED, the results in this study did not depend on self-rated questionnaires, but rather the medical assessment made by the physician. According to the criteria of SED, the patient has had at least 6 months of intensive stress before the diagnosis, making it a strong proxy for chronic stress. In this study we do not have any information about if or to what extent patients with depression and no SED diagnosis were exposed to chronic stress prior to their diagnosis. Moreover, the time periods including (wash-out, inclusion and follow up) were chosen because we had access to the database from 2010 until 2023 and, given the time it can take to develop, investigate, and diagnose both MCI and dementia, we prioritized an extended follow-up period. Additionally, given that many patients with chronic stress experience burnout symptoms for years [25] we also thought it was important to include a washout year before inclusion. Further, because the nature of the data doesn’t allow for more specific sub-typing of depression, we cannot standardize the ICD coding on that level.

### Future implications

Further exploration into the potential association between biomarkers, risk genes (e.g., APOE), and stress and stress-related biomarkers (such as cortisol and astrocyte-derived markers) and depression, are warranted. This is particularly relevant given the proposal that plasma biomarkers, such as phosphotau, neurofilament light chain protein (NfL) and glial fibrillary acidic protein (GFAP), may serve as routine diagnostic biomarkers in a near future [10]. Moreover, cognitive status sampled and followed over years should be included in future longitudinal studies.

Future studies should examine the possibility that symptoms of depression and/or chronic stress could be prodromal symptoms of dementia rather than risk factors in some cases. Additionally, other risk factors related to depressive symptoms, such as alcohol use and hearing loss, and other neurological disorders, such as Parkinson's disease and multiple sclerosis, could be investigated further.

## Conclusion

A diagnosis of depression was associated with an approximately threefold increased risk for later MCI and almost twofold increased risk for later AD. The risk increased further if the patients that also had experienced chronic stress as indicated by a recorded diagnosis of SED. The findings suggest that chronic stress and depression may be independent risk factors for dementia and together they may have an additive effect on the risk for later dementia.

## Data Availability

The authors of the present paper are willing to cooperate on research projects. Data is available from halsodata.rst@regionstockholm.se after ethical approval.

## Abbreviations

AD: Alzheimer disease
CSF: Cerebrospinal fluid
SED: Chronic stress-induced exhaustion disorder
CI: Confidence interval
MCI: Mild cognitive impairment
ORs: Odds ratios
VAL: Stockholm Regional Health Care Data Warehouse
